# Tissue Sodium Accumulation Induces Organ Inflammation and Injury in Chronic Kidney Disease

**DOI:** 10.3390/ijms24098329

**Published:** 2023-05-05

**Authors:** Yasuhiko Ito, Ting Sun, Hiroya Tanaka, Makoto Yamaguchi, Hiroshi Kinashi, Fumiko Sakata, Shunnosuke Kunoki, Yukinao Sakai, Takuji Ishimoto

**Affiliations:** 1 Department of Nephrology and Rheumatology, Aichi Medical University, Nagakute 480-1195, Japan; 2 Department of Nephrology, Nagoya University Graduate School of Medicine, Nagoya 464-0813, Japan; 3 Department of Nephrology, Nippon Medical School, Tokyo 113-8602, Japan

**Keywords:** sodium storage, TonEBP, inflammation, organ dysfunction, IL-6

## Abstract

High salt intake is a primary cause of over-hydration in chronic kidney disease (CKD) patients. Inflammatory markers are predictors of CKD mortality; however, the pathogenesis of inflammation remains unclear. Sodium storage in tissues has recently emerged as an issue of concern. The binding of sodium to tissue glycosaminoglycans and its subsequent release regulates local tonicity. Many cell types express tonicity-responsive enhancer-binding protein (TonEBP), which is activated in a tonicity-dependent or tonicity-independent manner. Macrophage infiltration was observed in the heart, peritoneal wall, and para-aortic tissues in salt-loading subtotal nephrectomized mice, whereas macrophages were not prominent in tap water-loaded subtotal nephrectomized mice. TonEBP was increased in the heart and peritoneal wall, leading to the upregulation of inflammatory mediators associated with cardiac fibrosis and peritoneal membrane dysfunction, respectively. Reducing salt loading by a diuretic treatment or changing to tap water attenuated macrophage infiltration, TonEBP expression, and inflammatory marker expression. The role of TonEBP may be crucial during the cardiac fibrosis and peritoneal deterioration processes induced by sodium overload. Anti-interleukin-6 therapy improved cardiac inflammation and fibrosis and peritoneal membrane dysfunction. Further studies are necessary to establish a strategy to regulate organ dysfunction induced by TonEBP activation in CKD patients.

## 1. Introduction

**Figure 1:**
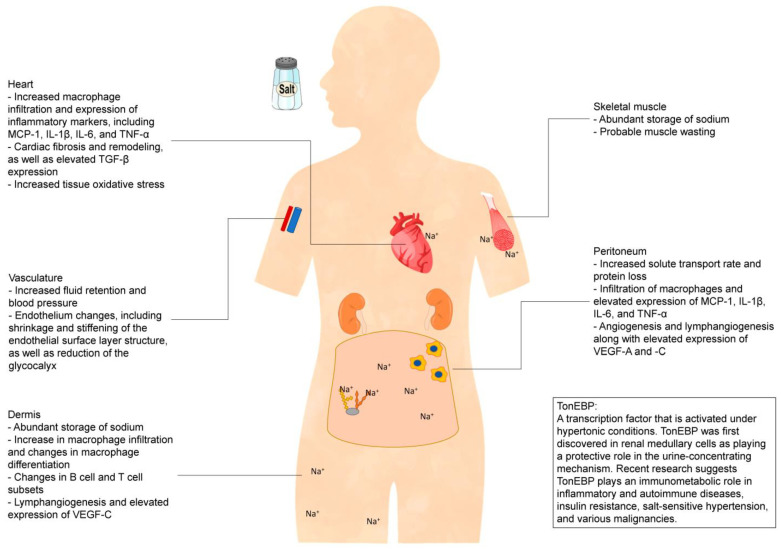
Excessive salt intake induces inflammation via local tonicity-responsive enhancer-binding protein (TonEBP) in the heart, vasculature, dermis, skeletal muscle, and peritoneum of patients with chronic renal failure.

Fluid overload is reportedly observed at high rates in chronic dialysis patients and in 30% of peritoneal dialysis (PD) patients [1,2,3,4]. One of the main causes of over-hydration in these patients is high salt intake. Sodium storage in tissues has recently emerged as an issue of concern [5]. Inflammation is a predictor of both all-cause and cardiovascular mortality in pre-dialysis chronic kidney disease (CKD) patients [6,7] and dialysis patients [6,8,9] and plays an important role in physiological conditions that lead to protein-energy wasting syndrome [10,11,12]. Predictors are usually measured in serum or plasma; however, the pathogenesis of the inflammation in CKD patients remains unclear. Therefore, current measures to treat these patients are insufficient. Many factors, such as cardiac preload, afterload, renin–angiotensin system activity, and oxidative stress, are involved in the development of inflammation and organ injuries in CKD. In this review, we focus on recent advances in research to elucidate the pathogenesis of tissue sodium accumulation driven by high-salt-intake-induced inflammation and organ injury in chronic renal failure (Figure 1).

## 2. Sodium Intake and Storage in CKD Patients and Healthy Individuals

The kidney plays an important role in the sodium in–out balance in humans. Sodium overload is a well-known feature of end-stage kidney disease, and it is associated with increased extracellular volume expansion, which leads to increased cardiovascular morbidity and mortality risks [13]. A study from the 1980s showed that, in normal individuals, the sodium balance is maintained even if they consume a high-salt diet. In contrast, sodium accumulation associated with increased blood pressure due to the impairment of sodium excretion was observed in patients with renal failure who consumed a high-sodium diet compared to healthy individuals [14]. In the general population, high salt intake is associated with hypertension, arteriosclerosis, heart failure, stroke, and CKD [15]. A population-based autopsy study indicated that a decreased estimated glomerular filtration rate (eGFR) is associated with cardiac hypertrophy and fibrosis [16]. In contrast to office and daytime systolic blood pressure, salt intake was found to be associated with only nocturnal blood pressure in non-dialysis CKD patients, which shows more predictive value in poor cardio-renal outcomes [17]. A low-salt diet tends to reduce the risk of renal events and adverse outcomes in patients with CKD [4,18,19,20,21,22,23]. The Kidney Disease Improving Global Outcomes guideline suggests that salt intake should be reduced to <2 g of sodium per day in CKD patients with high blood pressure [24]. The use of salt substitutes with added potassium, which have reduced sodium levels, can lower blood pressure and the risks of cardiovascular effects and death from any cause [25]. However, dietary sodium intake was much higher and potassium intake was much lower than the recommended levels in hypertensive subjects regardless of geographical area, as suggested by a national study of Italian populations [26].

In contrast to the traditional view of sodium homeostasis, recent studies have suggested that sodium excretion and retention are independent of blood pressure and body water [27]. Variability in 24 h sodium excretion was observed in healthy adults, even though total salt intake was fixed, thus providing evidence of tissue sodium storage. Rhythmic fluctuations in aldosterone, cortisol, and cortisone levels were found to be involved in this process [1,2,27]. Autocoids including endothelin-1 and adrenomedullin have also been demonstrated to be involved in sodium homeostasis. The renal excretion of endothelin-1 and adrenomedullin was found to be influenced by sodium intake, and, particularly, endothelin-1 was shown to play a role in salt-induced increases in glomerular pressure [28,29].

Recent studies have enabled the visualization of sodium accumulation in the lower extremities. ^23^Na magnetic resonance imaging (MRI) showed sodium storage in the interstitial tissue of the skin, subcutaneous tissues, and the muscles of the leg in disease conditions, which strongly supports the concept of sodium storage (Table 1) [30,31,32,33,34,35,36,37,38,39,40,41]. In non-CKD subjects, the sodium content in the calf muscles of men was age dependent [31,32]. In dialysis patients, the tissue sodium content was significantly higher than that in age-matched controls [32]. In this study, tissue sodium content was effectively reduced by 4 to 5 h of hemodialysis (HD) treatment. However, no relationship was found between measured sodium removed from plasma versus the tissues, suggesting that tissue sodium content varies during hemodialysis treatment.

In other studies that examined patients on maintenance HD or PD and controls with normal renal function, dialysis patients had higher skin and muscle sodium stores than the controls [32,35,36,37,38,39,41]. More recent studies have examined the significance of sodium storage in the tissues. In a study examining interleukin (IL)-8, E-selectin, and intracellular adhesion molecules, tissue sodium content was correlated with fluid overload and inflammation [34]. HD patients with cardiovascular disease had higher tissue sodium content than HD patients without cardiovascular disease. Tissue sodium accumulation in HD patients with cardiovascular disease progressed in parallel with higher plasma concentrations of the inflammatory marker IL-6 [39]. Skin sodium content was shown to be closely linked to left ventricular mass in patients with CKD [33]. Additionally, skin sodium levels were associated with all-cause mortality as an independent factor in patients on maintenance dialysis [41]. In PD patients, lower skin sodium content was associated with increased ultrafiltration, suggesting that skin Na^+^ quantification could be a predictor of peritoneal function [37]. These human studies using ^23^Na MRI suggest that sodium content in the skin is related to inflammation and associated with higher mortality risk. Strategies to prevent sodium accumulation and treatments to normalize the sodium content in tissues thus appear to be of high priority.

## 3. TonEBP Activation with and without Sodium Storage in Tissues

Signaling pathways involved in hypertonic conditions that are regulated by TonEBP have received considerable research attention with regard to tonicity-dependent and tonicity-independent sodium toxicity [1,5].

TonEBP, also known as the nuclear factor of activated T cell 5, was initially reported as an osmoprotective gene in renal medullary cells, which concentrate urine and are therefore always exposed to a high-osmotic environment [42,43,44]. To prevent apoptosis and the shrinkage of cells in the renal medulla and enable the adaptation to the hypertonic environment, poorly permeable organic solutes such as sorbitol, myo-inositol, taurine, betaine, glycerophosphorylcholine, or mannitol are produced by the upregulation of responsive genes via the TonEBP pathway [42,43,45,46,47,48,49]. Many types of non-renal cells, including heart and skeletal muscle cells, reportedly express TonEBP [50,51,52]. In addition, the biological importance of the osmotic stress response related to TonEBP-dependent gene regulation in non-renal tissues is now widely recognized [1,51,53]. High salt intake reportedly drives autoimmune diseases via TonEBP activation in immune cells [54,55].

Recent evidence has shown that TonEBP is regulated by various tonicity-independent factors as well as tonicity alone in both hypertonic and isotonic tissues [45]. Factors produced by mitochondria such as reactive oxygen species (ROS), microRNAs (miRNAs), epigenetic modifications, and viral infections have also been identified as regulators of TonEBP, and functional roles for these factors have been reported [45]. The stimulation of TonEBP upregulates the expression of pro-inflammatory cytokines such as tumor necrosis factor (TNF), C-C motif chemokine 2, IL-6, IL-1β, cyclooxygenase 2, and encoding inducible nitric oxide synthase and suppresses the expression of anti-inflammatory genes [44]. Many miRNAs that regulate TonEBP have been identified; however, most of them suppress TonEBP, resulting in the inhibition of target gene expression [56,57,58,59,60,61,62].

TonEBP plays roles in many disease conditions, such as cancer, cardiovascular disease, diabetes mellitus, brain diseases, and autoimmune diseases such as rheumatoid arthritis [42,44,63,64]. Mice deficient in the TonEBP gene exhibit renal medullary atrophy [65], nephrogenic diabetes insipidus, and pyelonephritis [66]. Inducible nitric oxide synthase production and T cell activation, which play roles in antimicrobial defense and immune responses, are also regulated via TonEBP [67,68,69]. TonEBP binds to the CD24 promoter in response to hypertonicity [68].

**Figure 2:**
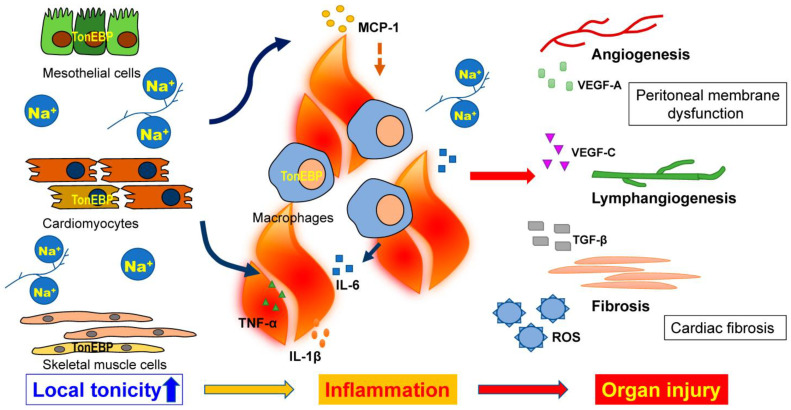
Under renal failure conditions with high salt intake, TonEBP plays a crucial role in the development of inflammation and tissue damage in the heart and peritoneal membrane. In 5/6Nx/NaCl mice with renal failure, high salt intake upregulates TonEBP in mesothelial cells and cardiomyocytes via increased local tonicity due to accumulation of sodium. Activation of TonEBP induces MCP-1 expression, leading to macrophage infiltration and upregulation of inflammatory cytokines, at least partly via the TonEBP–MCP-1 pathway. Inflammation causes tissue damage in the heart and peritoneal membrane, leading to cardiac fibrosis and high peritoneal transport rate with neoangiogenesis and lymphangiogenesis in PD.

High salt intake induces lymphangiogenesis in the skin via the TonEBP–vascular endothelial growth factor (VEGF)-C stimulation pathway (Figure 2) [70]. Lymphangiogenesis promoted by VEGF-C enhances tissue sodium removal, thus helping to decrease blood pressure, as demonstrated in animal models [2,70]. Similarly, human studies have reported a relationship between higher skin sodium content and lower circulating VEGF-C levels in populations with normal renal function [4]. A study examining tissue Na^+^ levels before and after hemodialysis treatment using ^23^Na MRI found a higher removal of sodium in muscle and skin by hemodialysis treatment in patients with higher circulating VEGF-C levels [32].

## 4. Salt-Induced Tissue Inflammation in Renal Failure Model Mice with Salt Loading

Studies related to salt-induced inflammation in renal failure models are summarized in Table 2 [50,71,72,73,74,75,76,77,78]. We collected articles related to renal failure models with salt loading published since 2010. We identified four articles focusing on the kidney [72,73,75,76], three articles regarding the heart [50,71,78], and two articles focusing on the cerebro–renal interaction [73,76]. Inflammation was reported in nine articles. Possible factors related to salt-induced inflammation included parathyroid hormone, asymmetric dimethylarginine, IL-6, the renin-angiotensin axis, oxidative stress, TonEBP, monocyte chemotactic protein-1 (MCP-1), nuclear factor-kappa B, and signal transducer and activator of transcription 1.

We previously reported the effects of high salt loading on tissue and systemic inflammation in subtotal nephrectomized (5/6Nx) mice, an adenine-induced renal failure model, and in cultured cells [50] (Figure 1). Macrophage infiltration was observed in the heart, peritoneal wall, and para-aortic tissues in 5/6Nx mice with 1% NaCl loading (5/6Nx/NaCl), but macrophages were not prominent in 5/6Nx mice administered tap water (5/6Nx/water), mice subjected to sham surgery with tap water (sham/water), and sham surgery mice with 1% NaCl loading (sham/NaCl) (Figure 1). No significant differences were detected in blood pressure or renal function between the 5/6Nx/NaCl and 5/6Nx/water mice. The measurement of sodium levels by chemical analysis indicated that sodium storage in the abdominal wall tissues was significantly higher in 4-week 5/6Nx/NaCl mice and 8-week 5/6Nx/NaCl mice compared to sham/water mice. Macrophage infiltration with the upregulation of inflammatory cytokine expression in 5/6Nx/NaCl mice was attenuated by diuretics or a change from salt water to tap water [50]. In these experiments, TonEBP mRNA expression was upregulated in the heart and peritoneal membrane in 5/6Nx/NaCl mice, and TonEBP translocation to the nucleus of cells in the heart was observed in 5/6Nx/NaCl mice and cultured mesothelial cells under high osmotic conditions [50,74].

In order to confirm the effects of salt loading under renal failure conditions, we investigated the pathophysiology in adenine-induced CKD model mice. Macrophage infiltration was greater in the peritoneal wall and heart in association with the upregulation of TonEBP and inflammatory cytokines induced by 1% NaCl drinking water in the adenine-induced CKD model when compared to adenine-induced CKD without salt loading [50], which was similar to the effects observed in 5/6Nx/NaCl mice. These findings indicate that a high-salt diet induces inflammation under conditions of renal dysfunction with salt loading.

## 5. Salt-Induced Cardiac Inflammation and Fibrosis in Renal Failure

A strong relationship exists between renal failure and cardiovascular disease, and lower eGFR and increased albuminuria are independently associated with higher cardiovascular disease mortality [79,80]. The adjusted hazard ratios of cardiovascular disease mortality were 9.5 for an eGFR of 15–29 mL/min/1.73 m^2^ and a urinary albumin-to-creatinine ratio (UACR) of ≥300 mg/g compared to the reference values of an eGFR of 90–104 mL/min/1.73 m^2^ and a UACR of <10 mg/g [80]. Against this backdrop, it has been reported that patients aged 30 years with stage 3B or 4 CKD have a reduction in life expectancy of 17 or 25 years, respectively, compared to individuals without CKD [81].

Generally, myocardial fibrosis is often initiated by the injury or death of cardiomyocytes, which results in increased extracellular matrix production by cardiac fibroblasts and myofibroblasts to repair the damaged tissues [82,83]. In the injured heart, the production of proinflammatory cytokines such as TNF-α, IL-1β, and MCP-1 upregulates the expression of profibrotic genes, especially transforming growth factor (TGF)-β1 [84]. TGF-β1 is a major profibrotic growth factor that amplifies fibrosis by promoting the expression of other profibrotic genes via Smad-dependent or Smad-independent pathways [85]. TGF-β1 also regulates cardiomyocyte growth and death, and the overexpression of TGF-β1 results in cardiomyocyte hypertrophy [86,87,88]. The process leading from inflammation to fibrosis results in systolic and diastolic dysfunction [89], and patients with CKD exhibit worse outcomes both in the short and long term compared to non-CKD patients [90,91]. As such, attenuating myocardial fibrosis has significant therapeutic value. Oxidative stress under ischemic or heart remodeling conditions also plays a role in the pathogenesis of fibrotic conditions. TGF-β stimulation accelerates mitochondrial ROS generation [92] and increases nicotinamide adenine dinucleotide phosphate oxidase (NOX) expression, which further elevates ROS levels in cells. ROS reportedly mediate angiotensin II-stimulated cardiac fibrosis [93,94]. Oxidative stress is induced in the uremic milieu and considered to be a major trigger of cardiovascular disease in CKD patients [95,96].

Left ventricular diastolic dysfunction is often observed in CKD patients, even in early stages, and it is associated with ventricular fibrosis and capillary loss [16,97]. In patients with CKD, a number of characteristic factors affect the progression of myocardial fibrosis, including hemodynamic overload; mechanical stress; uremic solutes (such as β2 microglobulin, indoxyl sulfate, *p*-cresol sulfate, and asymmetrical dimethylarginine); hormonal factors (norepinephrine and renin-angiotensin-aldosterone); the generation of ROS; metabolic (insulin resistance) and endocrine factors (Klotho deficiency, fibroblast growth factor 23, parathyroid hormone excess, and vitamin D deficiency); the CKD-related over-synthesis of molecules (advanced glycation end-products); and multifactorial growth factors (TGF-β1) [98,99]. However, details regarding the relationships and interactions between these factors and tissue sodium storage remain unclear.

**Figure 3:**
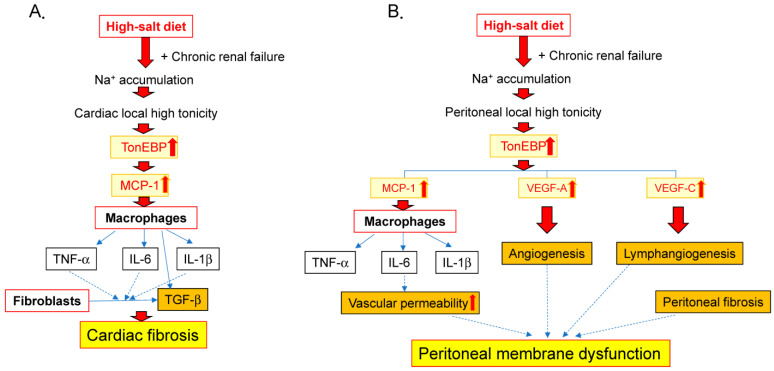
Mechanisms of tissue-sodium-accumulation-induced cardiac fibrosis and peritoneal membrane dysfunction via TonEBP activation. (**A**) Heart; (**B**) peritoneal membrane. (**A**) High salt intake induces activation of TonEBP, which upregulates MCP-1 expression by cardiomyocytes, leading to macrophage infiltration via the TonEBP–MCP-1 pathway in CKD [50]. Macrophages express inflammatory mediators such as TNF-α, IL-6, and IL-1, leading to induction of TGF-β in inflammation during the fibrosis process [78]. TonEBP plays an important role in cardiac inflammation and cardiac fibrosis in patients with renal failure and high salt intake. (**B**) Excessive dietary salt intake under renal dysfunction enhances accumulation of sodium, leading to activation of TonEBP [50]. TonEBP upregulates MCP-1, inflammatory cytokines, VEGF-A, and VEGF-C in vivo. IL-6 enhances MCP-1 and VEGF-A expression [74], leading to further macrophage infiltration and angiogenesis. High-tonicity conditions upregulate expression of MCP-1, IL-6, VEGF-A, and VEGF-C by mesothelial cells and macrophages via TonEBP activation [74]. TonEBP regulates inflammatory and angiogenetic changes in the peritoneum of CKD patients. These structural changes result in peritoneal membrane dysfunction.

High salt intake is related not only to elevated blood pressure but also endothelial dysfunction, arterial and ventricular stiffening, and ventricular hypertrophy and fibrosis [100]. The assessment of serum biomarkers of chronic inflammation could predict the development of cardiovascular disease, anemia, and sarcopenia, all of which contribute to high mortality in dialysis patients [8,101,102]. We demonstrated that the salt loading (1% NaCl in drinking water) of subtotal nephrectomized mice induces the upregulation of cardiac TonEBP expression and promotes macrophage infiltration in an MCP-1-dependent manner, leading to cardiac fibrosis (Figure 1, Figure 2 and Figure 3A) [50]. Serum and glucocorticoid-regulated kinase 1 (Sgk1) is a mineralocorticoid receptor-dependent gene associated with cardiac inflammation and fibrosis [103] as well as cardiac remodeling [104]. Reducing salt loading in mice by administering diuretics or changing 1% NaCl-containing water to tap water attenuated macrophage infiltration and the expression of inflammatory markers, including MCP-1 and Sgk1, and was associated with a reduction in TonEBP expression in the heart [50]. In addition, the upregulation of MCP-1 and Sgk1 expression induced by culturing cardiomyocytes in a high-osmotic-strength medium (Na 190 mEq/L) was suppressed by the addition of TonEBP small interfering RNA, indicating that both MCP-1 and Sgk1 are downstream of TonEBP (Figure 2) [50].

Although TonEBP is a transcription factor that connects osmotic stimuli with cardiac inflammation and fibrosis, conventional TonEBP-knockout animals reportedly die in the perinatal period, which makes it difficult to analyze TonEBP function in vivo [65,105,106]. Although a conditional-knockout mouse strain has been developed [107], the effect of TonEBP gene deletion on cardiac inflammation and fibrosis in these mice has not been reported. Evidence supporting the efficacy of therapeutic strategies targeting TonEBP in terms of heart disease is lacking. Therefore, we investigated whether treatment with an anti-IL-6 receptor antibody reduces cardiac inflammation and fibrosis in a mouse model of CKD with salt loading [78] as a possible target downstream of TonEBP. In the heart of subtotal nephrectomized mice with high salt intake, the expression of pro-inflammatory markers such as IL-6, TNF-α, and IL-1β was significantly upregulated, and F4/80-positive macrophage infiltration was significantly increased (Figure 2). Furthermore, cardiac fibrosis and hypertrophy were significantly exacerbated in the heart of renal failure mice with high salt intake (Figure 2). Cardiac oxidative stress, as indicated by the expression of mRNA-encoding NOX-2, was increased in the heart of subtotal nephrectomized mice with high salt intake. Treatment with an anti-mouse monoclonal IL-6 receptor antibody significantly ameliorated cardiac inflammation, fibrosis, and partial oxidative stress in the heart of subtotal nephrectomized mice without lowering blood pressure [78]. However, the upregulation of TonEBP was not suppressed by anti-IL-6 receptor antibodies, indicating that this strategy improved the effects downstream of TonEBP and did not modulate local tissue tonicity [78]. These results suggest that anti-IL-6 therapy could inhibit the development of cardiovascular disease caused by high salt intake in CKD patients. Tissue sodium accumulation was shown to be correlated with plasma IL-6 levels in HD patients with cardiovascular disease progression [39]. Indeed, a clinical study in which anti-IL-6 drugs were administered for the purpose of treating cardiovascular disease demonstrated that biomarkers of inflammation and thrombosis were decreased in CKD patients [108]. In these respects, IL-6 could be targeted as a means of reversing cardiac inflammation and fibrosis related to high salt intake.

A high-salt diet was shown to induce interstitial sodium accumulation and inflammatory cell infiltration in the skin of rats [70]. Other recent studies indicated that skin sodium concentration is correlated with a left ventricular mass in CKD patients (Figure 1). These findings are in agreement with previous animal studies [50,67]. Although it remains unclear whether skin sodium concentration is correlated with cardiac sodium concentration, our previous study demonstrated that cardiac TonEBP expression is enhanced by high salt intake [50]. Direct measurements of cardiac sodium concentrations using MRI are technically challenging at present [109].

Further studies are necessary to elucidate the detailed mechanism by which high salt intake promotes cardiac inflammation and whether anti-inflammatory therapy is an efficacious treatment option for addressing cardiovascular disease in CKD patients. In addition, efforts to identify other strategies to ameliorate inflammation induced via the TonEBP activation pathway would be worthwhile.

## 6. Salt-Induced Inflammation in the Peritoneum during Renal Failure

PD is an effective renal replacement therapy approach utilized worldwide due to its good outcomes. During the last decade, PD has become more common in a number of countries, including China, Thailand, and the United States [110]. As such, protecting the peritoneal structure and function in patients with CKD is important, even in patients in the pre-dialysis phase.

Excessive salt intake is closely associated with fluid retention and hypertension in dialysis patients [2,15]. In continuous ambulatory PD patients, a linear correlation was observed between achieved ultrafiltration failure and sodium removal [111]. Thus, in PD patients, high salt intake is thought to increase the need for ultrafiltration and the use of hypertonic-glucose PD fluids, which in turn directly causes peritoneal damage [112].

The potential for direct sodium toxicity in PD has gained increased interest in recent years. At the time of PD initiation, the baseline peritoneal solute transport rate (PSTR) varies, and a correlation exists between high PSTR and a poor prognosis in PD due to inadequate ultrafiltration [113]. Factors such as gender, age, genetics, and diabetes are thought to be related to baseline PSTR. The presence of IL-6 in dialysate is closely related to high baseline PSTR. In addition, a positive relationship was observed between dialysate IL-6 and dialysate MCP-1 levels as well as VEGF-A [114,115]. Notably, a pathological analysis of peritoneal tissues in pre-dialysis patients identified local peritoneal inflammation and vascular density as predictors of baseline peritoneal permeability. The frequencies of CD68-positive macrophages, IL-6-positive cells, and CD31-positive blood vessels were shown to be correlated with the baseline dialysate–plasma ratio of creatinine and the baseline peritoneal transport rate [116].

Animal experiments have suggested that excessive salt intake affects the peritoneal microenvironment and thus peritoneal function. In rats with normal renal function, a high-salt diet induced the epithelial-to-mesenchymal transition of the peritoneal membrane and promoted peritoneal fibrosis with the upregulation of TGF-β1 and IL-6 mRNA expression [117]. In uremic mice with high salt intake, higher blood vessel and macrophage density, as well as higher PSTR, were observed compared to uremic mice without high salt intake or normal mice with high salt intake. We previously observed a significant increase in sodium storage in the abdominal wall tissues [50]. Local increases in tonicity activate the transcription factor TonEBP, which leads to the local upregulation of inflammatory and pro-angiogenic mediators in the peritoneum, including IL-6, MCP-1, VEGF-A, and VEGF-C. TonEBP may thus play a crucial role in the process of peritoneal deterioration induced by sodium accumulation (Figure 2 and Figure 3) [50,74].

In cultured Met 5A mesothelial cells and RAW 246.7 macrophages, a high-sodium medium (Na: 190 mEq/L) was shown to stimulate the expression of TonEBP as well as VEGF-A, VEGF-C, and IL-6. The suppression of TonEBP by small interfering RNA downregulated the overexpression of these mediators, suggesting that TonEBP plays a key role during the process of peritoneal inflammation and hyperpermeability [74]. The blockade of IL-6 by a monoclonal anti-IL-6 antibody, MR16-1, was found to effectively alleviate local peritoneal inflammation induced by high salt intake in uremic mice. An improvement of peritoneal ultrafiltration function as well as a lower solute transport rate was observed in mice treated with MR16-1 [74].

In mice with the myeloid-specific deletion of the TonEBP gene, peritoneal macrophages showed a reduced expression of TNF-α and iNOS when exposed to lipopolysaccharide. NFκB activation was enhanced by increased TonEBP expression in RAW264.7 macrophages [49]. In addition to macrophages, elevated TonEBP expression stimulated by high osmolality induced by high glucose led to increased MCP-1 expression in human peritoneal fibroblasts and mesothelial cells in vitro. It was hypothesized that this process is NFκB-dependent [118,119]. Determining whether high sodium induces the same effect requires further study.

The differentiation of T-helper (Th) cell subsets reportedly regulates peritoneal immune responses during PD. Th17 cells are highly proinflammatory and secrete cytokine IL-17A, which is reportedly involved in various chronic inflammatory diseases. IL-17A was found to drive peritoneal inflammation and fibrosis in animal models [120]. Exposing T cells to a high-sodium environment promoted Th17 cell polarization in experimental autoimmune encephalitis [54]. Determining whether sodium accumulation in the peritoneum promotes Th17 cell differentiation and IL-17 excretion may be a future research project.

Sodium is thought to accumulate in soft tissues primarily in an osmotically inactive form. Glycosaminoglycans (GAGs) are thought to play a crucial role in sodium regulation in soft tissues by binding free sodium ions. The sodium in soft tissues appears to be osmotically inactive, as demonstrated in several animal studies [121,122]. Both human and animal studies have found that GAG expression varies in tissues such as the skin, muscle, arteries, and myocardium, and this variation in GAG expression is correlated with tissue sodium content [123,124]. However, a recent high-salt-diet animal model study found that sodium ions are stored intracellularly in the dermis as well as in the muscle and myocardium under high-salt conditions without additional hydration; this storage was independent of the GAG level in the tissues [125]. Of course, the upregulation of TonEBP suggests that sodium ions are released by GAGs. Sodium ions are shifted from the extracellular to the intracellular compartment, and local tonicity appears to be controlled by GAGs [125]. This creates locally high interstitial tonicity that regulates macrophage and T cell differentiation, thereby affecting various immune responses [67].

The vascular endothelium is the first barrier encountered in the transport of sodium from the blood to tissues. The endothelial surface layer (ESL) of blood vessels is abundant in GAGs, which are thought to play a role in sodium homeostasis [126]. Negatively charged ESLs appear to contain osmotically inactive sodium ions [127]. In vitro studies showed the shrinkage and stiffening of the ESL under high-sodium conditions. A reduction in heparan sulfate of up to 68% was observed with 5 days of sodium overload treatment, concomitantly with the transport of sodium into endothelial cells. These studies showed that high-sodium conditions can injure endothelial cells as well as the ESL, which could facilitate the entry of sodium into endothelial cells and interstitial tissues (Figure 1) [128,129]. Heparan sulfate constitutes the majority of GAG in the endothelial glycocalyx [130]. The endothelial glycocalyx is thought to be damaged in renal failure, leading to the shedding of its constituents into the circulation [131,132,133,134]. The impairment of the ESL also occurs in hypertension and is associated with vascular stiffness and myocardial dysfunction [135]. A decline in NO release is observed during this process, which is believed to be an important factor during the progression of heart failure [128,136].

A pathological study of the peritoneum found a greater preservation of fucose-containing sugar chains and heparan sulfate in the peritoneal vasculature in patients treated with pH-neutral PD fluid, and the PSTR of these patients was better than that of patients treated with conventional acidic PD solution [137]. Therefore, it could be interesting to further explore the relationship between salt intake and changes in the ESL and glycocalyx in the peritoneal vasculature.

An animal study showed that diminished peritoneal function could be reversed in uremic mice after removal from a high-salt diet, concomitantly with reductions in IL-6 and VEGF-A levels in the dialysate [74]. From the perspective of volume overload, it is also beneficial to restrict salt intake in PD patients. However, other studies have made different conclusions regarding salt intake in dialysis patients. A single-center, retrospective cohort study identified low dietary sodium intake as an independent predictor of overall and cardiovascular mortality in PD patients. The same study reported that patients with high average salt intake are more likely to be younger, male, overweight, and with more nutrient intake paralleling sodium intake [138]. Similar findings were also demonstrated in Japanese hemodialysis patients [139,140]. As sodium accumulation varies by individual, the ideal level of sodium intake in both normal populations and patients with renal failure remains controversial.

## 7. Strategy to Ameliorate Inflammation Induced by Sodium Storage-Related TonEBP Stimulation in Renal Failure

In our study, inflammation with the upregulated expression of pro-inflammatory cytokines was reversed in the heart, abdominal wall, and para-aortic tissues of 5/6Nx mice with salt loading by the administration of diuretics or a change from high-salt drinking water to tap water [50]. These findings suggest that educating and encouraging patients with CKD to adopt a salt-restricted diet is important, even after starting dialysis therapy. The identification of high-salt habits among hypertensive patients, which could be assessed using a short questionnaire, is reportedly important to reduce mortality for cardiovascular diseases [141].

Increased dietary potassium can lower sodium sensitivity in both normotensive and hypertensive populations [142]. It is widely believed that a high-potassium diet can lead to hyperkalemia in patients with renal failure. Considerable evidence suggests that higher baseline serum potassium levels are associated with a higher risk of cardiovascular mortality [143,144]; however, an association between food-derived potassium and all-cause or cardiovascular mortality has not been established. Two prior longitudinal studies of potassium intake in HD patients showed conflicting results [145,146]. In a recent study of 8043 HD patients, higher dietary potassium intake was not associated with hyperkalemia and all-cause or cardiovascular mortality [147]. Potassium depletion reportedly enhances renal sodium pump activity, thus promoting sodium retention [148]. These findings suggest that potassium intake may have a beneficial effect in CKD patients. Future studies should examine the effect of potassium intake on tissue sodium accumulation and establish safe levels of potassium intake for CKD patients.

In addition to dietary sodium restriction, some medications are also believed to decrease tissue sodium deposition. In a study of acute heart failure using ^23^Na MRI, sodium accumulation was found in muscle and skin. Intravenous furosemide therapy resulted in a reduction in sodium deposition in the skin in all nine patients studied and in the muscle of seven of the nine patients [149]. In animal experiments, furosemide effectively suppressed inflammation in the heart and peritoneal and para-aortic tissues in high-salt-intake renal failure mice [50], suggesting that furosemide is useful for preventing sodium-accumulation-induced inflammation. In another study involving patients with type 2 diabetes, treatment with the sodium-glucose cotransporter 2 inhibitor dapagliflozin for 6 weeks decreased sodium content in the skin and muscles of the lower leg, as determined by ^23^Na MRI [150]. Further studies are necessary to establish optimal medications to treat tissue sodium storage in CKD patients.

Organ damage such as cardiac fibrosis and peritoneal membrane dysfunction can be improved by anti-IL-6 treatment [74,78]. Recent studies have indicated that anti-inflammatory therapies targeting IL-1 or IL-6 are promising for improving the outcome of CKD patients with inflammation [108,151,152,153,154] (Table 3). Anti-IL-6 antibody treatment was shown to ameliorate inflammation and renal anemia associated with iron metabolism in hemodialysis patients with inflammation [151]. Data from animal experiments and these clinical trials suggest that strategies involving anti-inflammatory treatments such as anti-IL-6 receptor monoclonal antibodies can prevent cardiac inflammation and fibrosis in the clinical setting. Thus, further human studies examining the efficacy of anti-IL-6 antibodies to prevent cardiac inflammation and fibrosis are warranted.

Of course, dietary salt restriction is the basis of treatment to prevent high-salt-induced organ damage in renal failure. In addition, a clinical trial of a low-sodium dialysate for PD is underway in Japan. The effectiveness of sodium removal using a low-sodium dialysate has been reported in PD and HD [155,156]. Thus, a new hyponatremic dialysate solution for patients on PD is expected.

**Table 3:** Therapeutic research using biologics targeting inflammation in subjects with chronic kidney disease.

Human/ Mouse	Number of Subjects	Agent	Outcome	Ref.
human	22 maintenance HD patients	Anakinra (IL-1 receptor antagonist)	Significant increase in serum CRP and IL-6 levels	[152]
human	22 maintenance HD patients	Anakinra (IL-1 receptor antagonist)	Significant increase in serum adiponectin levels	[157]
human	42 CKD stage 3 and 4 patients	Rilonacept (IL-1 trap)	Significant decrease in brachial artery FMD, the assessment of endothelial function	[154]
human	61 maintenance HD patients	Ziltivekimab (anti–IL-6 antibody)	Significant increase in serum albumin and decrease in ERI and ESA usage	[151]
human	264 maintenance HD patients	Ziltivekimab (anti–IL-6 antibody)	Significant decrease in serum hsCRP, fibrinogen, haptoglobin, SAA, sPLA2, lipoprotein (a),	[108]
mouse	32 mice with acute kidney injury after IRI	Etanercept (TNF-α antibody)	Significant decrease in kidney fibrosis of about 25% and blockade of AKI-to-CKD transition	[158]

Abbreviations: acute kidney injury (AKI), chronic kidney disease (CKD), C-reactive protein (CRP), erythropoiesis-stimulating agent resistance index (ERI), erythropoiesis-stimulating agent (ESA), flow-mediated dilation (FMD), hemodialysis (HD), high-sensitivity C-reactive protein (hsCRP), interleukin (IL), ischemia reperfusion injury (IRI), serum amyloid A (SAA), secretory phospholipase A2 (sPLA2), tumor necrosis factor (TNF)-α.

## 8. Conclusions

High salt intake induces tissue inflammation associated with organ dysfunction in CKD patients. TonEBP appears to play a crucial role in the process of cardiac fibrosis and peritoneal deterioration induced by sodium overload. The suppression of these pathways is important for improving morbidity and mortality in CKD patients. However, research from this perspective is still in its infancy. Further investigations of the pathogenesis of organ injury induced by salt accumulation in renal failure are needed. Further studies are also necessary to establish a strategy to regulate inflammation and related organ dysfunction induced by TonEBP activation in CKD patients.

## Figures and Tables

**Table 1:** Studies of CKD or cardiovascular disease evaluated using ^23^Na MRI.

Year	Study Design	Subjects	Findings	Refs
2012	Cross-sectional	Normotensive subjects (17 men, 13 women) and 5 patients with primary aldosteronism	29% increase in muscle sodium content in patients with aldosteronism compared with normal subjects.	[30]
2013	Cross-sectional	56 healthy control men and women, 57 men and women with essential hypertension	Age-dependent increases in sodium content was observed in calf muscle in men. Patients with refractory hypertension showed increased tissue sodium content compared with normotensive controls.	[31]
2015	Cross-sectional	24 HD patients and 27 age-matched healthy controls, 20 HD patients before and shortly after HD	Age was associated with higher tissue sodium content in controls. Older HD patients showed increased sodium and water in skin and muscle compared with age-matched controls. After HD, patients with low circulating VEGF-C levels showed higher skin sodium content compared with HD patients with high VEGF-C levels.	[32]
2017	Cross-sectional	99 patients with mild to moderate CKD (42 women)	Skin sodium content, but not total overhydration, correlated with systolic blood pressure. Skin sodium content was closely linked to left ventricular mass in CKD patients.	[33]
2019	Cross-sectional	23 patients with CKD5, 11 healthy controls	CKD patients had fluid overload when compared to controls. Tissue sodium concentrations were higher in the subcutaneous compartment, but not in the muscle. Tissue sodium content was correlated with fluid overload. Fluid overload in CKD was linked to higher IL-8 and inversely associated with E-selectin. Higher subcutaneous sodium concentration was linked to higher ICAM.	[34]
2020	Cross-sectional	10 healthy controls, 20 patients with CKD5 (not on dialysis)	CKD patients had higher sodium and lower extracellular resistance. Tissue sodium concentration has an inverse linear relationship with extracellular resistance.	[35]
2020	Cross-sectional	10 healthy controls, 12 CKD3-5, 13 HD, 10 PD patients	Tissue sodium in the skin, sleus, and tibia was higher in HD and PD patients compared with controls. Serum albumin showed a negative correlation with soleus sodium in HD patients. Estimated GFR showed a negative correlation with tissue sodium in merged control-CKD patients. Hemoglobin was negatively correlated with tissue sodium concentration in CKD and HD patients.	[36]
2021	Cross-sectional	162 subjects (10 PD, 33 HD patients, 119 controls)	Patients on PD and HD showed higher muscle and skin sodium accumulation compared with controls. African American patients, older age, and male sex were associated with increased sodium content. Greater ultrafiltration was associated with lower skin sodium content in PD patients. Higher plasma IL-6 and hsCRP levels correlated with increased muscle and skin sodium content in the subjects.	[37]
2021	Prospective	18 patients with HF, 34 HD patients, 31 patients with CKD (GFR matched to the HF patients)	HF patients showed higher skin sodium content than matched CKD patients, which was indistinguishable from skin sodium content in HD patients.	[38]
2022	Cross-sectional	52 HD patients divided into groups with (23 subjects) or without (29 subjects) a positive history of cardiovascular events	HD patients with previous CVD events showed an increased sodium content in skin and muscle tissue compared to HD patients without CVD events. Fluid amount was not different between groups. Tissue sodium accumulation in HD-CVD patients was paralleled by higher plasma IL-6 levels.	[39]
2022	Cross-sectional	36 pediatric participants (17 healthy, 19 CKD) and 19 healthy adults	Healthy adults had higher tissue sodium content compared with pediatric groups. No differences in tissue sodium content were found between healthy children and CKD patients. CKD patients with glomerular disease showed increased sodium content; however, CKD patients with tubular disorders showed reduced sodium content.	[40]
2022	Prospective	52 subjects (10 PD, 42 HD patients)	Higher skin sodium accumulation was associated with worse clinical outcomes. Skin sodium concentration could be a predictor of survival.	[41]

**Table 2:** Studies related to salt-induced inflammation in renal failure models.

Year	Model	Findings	Refs
2011	Subtotal nephrectomized rats treated with NaCl drinking water	NaCl-loaded subtotal nephrectomy caused LVH, hypertension, and increased plasma levels of PTH, creatinine, inorganic phosphorus, ADMA, and IL-6.	[71]
2013	5/6 nephrectomized rats with nitric oxide depletion and a high-salt diet	Renal failure developed by 12 weeks after subtotal nephrectomy. Inflammation-related tubulo-interstitial damage and fibrosis, tubular atrophy, and focal glomerulosclerosis led to massive reduction of healthy glomeruli within the remnant kidney.	[72]
2015	5/6 nephrectomized rats with high-salt diet	High salt activated the intrarenal and cerebral renin-angiotensin axes, which is related to the promotion of oxidative stress, renal fibrosis, and progression of CKD.	[73]
2017	5/6 nephrectomized mice with salt loading	Salt loading induced macrophage infiltration in the peritoneal wall, heart, and para-aortic tissues through the TonEBP–MCP-1 pathway.	[50]
2019	5/6 nephrectomized mice with salt loading	Salt loading induced peritoneal inflammation, angiogenesis, and high peritoneal transport rate. Blockade of IL-6 signaling rescued peritoneal transport function.	[74]
2020	Rats treated with nitric oxide synthase inhibition and a high-salt diet	Early treatment with NF-κB inhibitor prevented the development of late renal injury and inflammation.	[75]
2021	High-salt-load 5/6 nephrectomized rats	Inhibition of central PRR expression reduced inflammation and oxidative stress in both brain and kidney and ameliorated renal injury and fibrosis. The central MAPK/ERK1/2 and PI3K/Akt signaling pathways were related to the mechanism as well as the angiotensin-converting enzyme 1–angiotensin II–angiotensin type 1 receptors axis.	[76]
2021	5/6 nephrectomized rats with NaCl treatment	NaCl treatment induced a pro-inflammatory phenotype in peritoneal macrophages derived from nephrectomized rats. High salt intake promoted immune activation of macrophages through phosphorylation of STAT1.	[77]
2022	5/6 nephrectomized mice with salt loading	Salt loading induced cardiac inflammation and fibrosis along with elevated levels of proinflammatory cytokines. Blockade of IL-6 signaling showed anti-inflammatory, antifibrotic, and partial antioxidative effects on the heart.	[78]

Abbreviations: left ventricular hypertrophy (LVH), parathyroid hormone (PTH), asymmetric dimethylarginine (ADMA), interleukin-6 (IL-6), chronic kidney disease (CKD), tonicity-responsive enhancer binding protein (TonEBP), monocyte chemotactic protein-1 (MCP-1), nuclear factor-kappa B (NF-κB), (Pro)renin receptor (PRR), mitogen-activated protein kinase (MAPK), extracellular signal-regulated kinase (ERK), phosphatidylinositol-3 kinase (PI3K), signal transducer and activator of transcription 1 (STAT1).

## Data Availability

Not applicable.

## Abbreviations

CKD	chronic kidney disease
ESL	endothelial surface layer
eGFR	estimated glomerular filtration rate
GAG	glycosaminoglycan
IL	interleukin
MCP-1	monocyte chemoattractant protein-1
miRNA	microRNA
MRI	magnetic resonance imaging
Nx	subtotal (5/6) nephrectomy
Nx-rat IgG1	rat IgG1-treated Nx mice with salt loading
Nx-MR16-1	MR16-1-treated Nx mice with salt loading
PD	peritoneal dialysis
PSTR	peritoneal solute transport rate
Sgk1	serum and glucocorticoid-regulated kinase 1
5/6Nx	sub-total-nephrectomized mice
5/6Nx/NaCl	5/6Nx with 1% NaCl loading
5/6Nx/Water	5/6Nx with tap water administration
Sham/Water	sham surgery with tap water
TGF-β	transforming growth factor-beta
TNF-α	tumor necrosis factor-alpha
TonEBP	tonicity-responsive enhancer-binding protein
UACR	urinary albumin-to-creatinine ratio
VEGF-A	vascular endothelial growth factor A
VEGF-C	vascular endothelial growth factor C
